# Green Deal and Circular Economy of Bottom Ash Waste Management in Building Industry—Alkali (NaOH) Pre-Treatment

**DOI:** 10.3390/ma15103487

**Published:** 2022-05-12

**Authors:** Nikolina Poranek, Beata Łaźniewska-Piekarczyk, Lidia Lombardi, Adrian Czajkowski, Magdalena Bogacka, Krzysztof Pikoń

**Affiliations:** 1 Department of Technologies and Installations for Waste Management, Faculty of Energy and Environmental Engineering, The Silesian University of Technology, Konarskiego 18, 44-100 Gliwice, Poland; magdalena.bogacka@polsl.pl (M.B.); krzysztof.pikon@polsl.pl (K.P.); 2 Department of Building Engineering and Building Physics, Faculty of Civil Engineering, The Silesian University of Technology, Akademicka 5, 44-100 Gliwice, Poland; 3 Doctoral School, The Silesian University of Technology, Akademicka 2A, 44-100 Gliwice, Poland; 4 Faculty of Engineering, Niccolò Cusano University, Via Don Carlo Gnocchi, 3, 00166 Rome, Italy; lidia.lombardi@unicusano.it; 5 Department of Power Engineering and Turbomachinery, Faculty of Energy and Environmental Engineering, The Silesian University of Technology, Konarskiego 18, 44-100 Gliwice, Poland; 6 EnergySol s.c., Przepiórek 53, 43-100 Tychy, Poland

**Keywords:** secondary waste, municipal solid waste incineration bottom ash, circular economy, European Green Deal, NaOH pre-treatment, heavy metals immobilization

## Abstract

This study aims to investigate the possibilities of municipal waste incineration bottom ash (MSWIBA) utilization in the construction sector. MSWIBA development fits into the European Green Deal, Sustainable Development Goals (SDGs), and the Circular Economy (CE). This manuscript describes current MSWIBA treatment such as solidification, ceramization, vitrification, chemical activation (NaOH, CaOH_2_, NA_2_SiO_3_ + NaOH, Na_2_CO_3_ + NaOH, NH_4_OH), acid treatment with diluted solutions (HCl, H_2_SO_4_), chemical stabilization (FeSO_4_, PO_4_^3−^), chelation, etc. For the purpose of comparative research, MSWIBA before valorization, after valorization, and after NaOH pre-treatment was investigated. In terms of their physico-chemical properties, the tested samples were examined. Three kinds of MSWIBA were used as a substitute for 30% of cement in mortars. The mortars were tested for 28-day strength. Leachability tests were performed in acid, aggressive, alkali, and neutral water environments. Life Cycle Assessment (LCA) analysis was carried out, which presented the environmental benefits of MSWIBA management in construction.

## 1. Introduction

**Figure 1:**
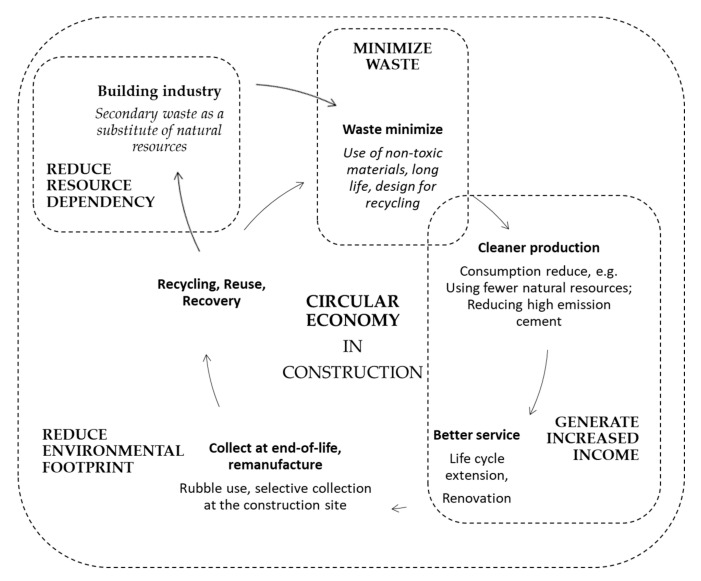
Circular economy in construction.

In the United Nations Framework Convention of 1992, a position was adopted stating the danger of raising the average global temperature by 2 °C (or even 1.5 °C) above pre-industrial levels [1]. The solution to preventing an increase in temperature is the development of the circular economy and the departure from the current linear economy. According to the waste management hierarchy, products and materials are re-used and recycled in an ideal circular economy. Their life cycle is consistently extended and environmental pollution and greenhouse gas emissions are reduced [2,3]. Figure 1 shows the circular economy in construction.

To improve the condition of the environment, the European Union introduced the European Green Deal [4]. The overarching goal of the European Commission’s policy initiatives is to achieve climate neutrality in Europe by 2050. The Green Deal addresses challenges such as clean energy supplies, protection and restoration of the natural environment, sustainable development and use of natural resources, and improving human health. The goal of the Green Deal is to build and implement a framework for responsible, sustainable behavior and use of the natural environment [5,6]. From an environmental point of view, the most important Green Deal initiatives are related to: Transition to a Circular Economy; Achieving Climate Neutrality; Clean, Reliable and Affordable Energy; and A Zero Pollution Europe. Each initiative has a strategy, a directive, and a specific course of action [7,8,9].

The green deal is integrated within the sustainable development goals (SDGs) adopted by the United Nations. The management of secondary waste in construction is in line with SDG 12 (Ensure sustainable consumption and production patterns) and SDG 13 (Take urgent action to combat climate change and its impacts), and can extend the life cycle of products or materials [7,10].

The use of secondary waste as a building material can contribute to achieving climate neutrality and lower pollution in Europe [11]. Using waste as a cement substitute is associated with lower greenhouse gas emissions (mainly CO_2_) and reduced extraction of natural resources. Emissions related to the extraction, processing, and use of energy are avoided. To produce a ton of cement, almost one ton of CO_2_ (dependent on technology) is produced [12].

The present research aims to analyze the possibility of recycling secondary waste from incineration plants. This is in line with the New Circular Economy Action Plan of closing the loop, which Figure 1 shows [8].

### 1.1. Current Market Situation with Residual Waste and Secondary Waste

The last element of a circular economy is energy recovery. Combustion of mixed (non-recyclable) municipal solid waste (MSW) is part of Clean, Reliable, and Affordable Energy. MSW that cannot be recycled is landfilled, but this is not part of the circular economy [13,14].

An exemplary installation with a capacity of 210,000 tons per year and an average calorific value of incinerated waste at the level of 7.8 GJ/Mg (GJ/ton) produces 300.370 GJ of thermal energy and 405.166 GJ of electricity annually [12,15].

The combustion of MSW in incineration plants generates more than 30% of secondary waste concerning the inlet stream. The main residue is MSW incineration bottom ash (MSWIBA), which accounts for ca. 90% of MSW incineration solid residues. About 18 million tons are produced annually in Europe even with efforts to reduce waste incineration; hence. the amount of MSWIBA is much greater worldwide, such as in China, Japan, and Singapore [16]. MSWIBA is heterogeneous waste and it depends directly on input material (MSW). The raw MSWIBA consists mainly of melt components and stones (approx. 81%), pieces of glass (approx. 17%), porcelain (approx. 0.5%), metals (approx. 0.07%), and trace amounts of unburned organic material. The composition of the MSWIBA is variable and depends on the season, the location of the incineration plant, and even the day [12].

MSWIBA is characterized by compatibility with high elasticity in its uncured state. In the construction industry, MSWIBA, thanks to its properties, can replace natural aggregate and be used as a construction material. However, to be used in the construction industry, MSWIBA should be subjected to valorization and appropriate processes. Fresh MSWIBA is metastable and strongly alkaline reactive (e.g., it contains lime, and anhydrite). One of the methods of safe MSWIBA management, compliant with the Reference Document on the Best Available Techniques for Waste Incineration, is its valorization, generally achieved by the steps described in the following manuscript [12].

To improve the quality of MSWIBA as a substitute for natural aggregate, sand, or cement, the concrete matrix can be compacted or different activation can be used, for example: solidification, ceramization, keramization, vitrification, chemical activation (NaOH, CaOH_2_, NA_2_SiO_3_ + NaOH, Na_2_CO_3_ + NaOH, NH_4_OH), acid treatment with dilute solutions (HCl, H_2_SO_4_), chemical stabilization (FeSO_4_, PO_4_^3−^), chelation, and other technologies depending on the needs, which are determined by the physicochemical composition and intended use of the end product. Ceramization and vitrification are high temperature processes and therefore are expensive, energy intensive, and non-environmentally friendly. Stabilization, solidification, and storage do not provide for recovery, and hence, do not fit into the circular economy. Other processes are still new, little known, and relatively expensive. This is why a market gap still exists; pilot and laboratory research is needed to fill this gap [8]. The MSWIBA is first cooled with water. Periodic storage of MSWIBA takes place under a roof or in a bunker for 1–3 months (sometimes the MSWIBA maturation/aging can take up to a year). The aging process causes hydration of metal oxides and oxidation of metallic Fe/Al. Then, the MSWIBA is divided into appropriate fractions (e.g., 0–40 mm and 40–150 mm fractions) by a rotary drum, and ferrous and non-ferrous metals are separated using magnetic separators. In this form, the MSWIBA matures further to allow the hydration processes to take place. The maturation process improves the physicochemical properties of the MSWIBA and reduces the leaching of heavy metals.

Bottom ash (BA, European Waste Codes: 19 01 12) is partly used as a priming method in the building industry [11]. Currently, the valorization process aims at improving physicochemical properties. The process consists in lowering the MSW incineration bottom ash (MSWIBA) temperature to 80–90 °C. Then, the MSWIBA is divided into 0–40 mm and 40–150 mm fractions in a rotary drum. The fractions are cleaned of ferrous and non-ferrous metals. The recovered metals are recyclable. Then, the fractions are prepared according to the recipient’s order (e.g., 0–8 mm and 8–40 mm). The pure fraction is aged under a roof to stabilize it. MSWIBA serve as ballast in a landfill as well [16,17].

Even after the described valorization process, the MSWIBA still contains mobile pollutants, e.g., heavy metals and salts, which are harmful to the environment. In addition, it contains substances that may have a negative effect on the concrete mix, such as metallic aluminum, glass, sulfur, chloride, and gypsum. Thus, using “raw” MSWIBA as a substitute for natural aggregate would produce a material with worse physical and chemical properties than the conventional one. Cracks and swelling are often noticed in concrete samples with the addition of MSWIBA due to its metallic Al or Al/Zn alloy content [18].

One possibility to reduce these adverse effects is to pre-treat the raw MSWIBA with NaOH. Equations (1) and (2) present the reactions of undesired Al and Zn, for instances, with NaOH:

2Al + 2NaOH + 6H_2_O → 2Na[Al(OH)_4_] + 3H**_2_**↑ pH > 11.75 (1)

Zn + 2NaOH + 2H_2_O → Na_2_Zn(OH)_4_ + H**_2_**↑ pH > 12 (2)

NaOH pre-treatment reduces the leachability of MSWIBA impurities (e.g., As, Ba, Cr, Cu, Pb, Ni, Se, Zn, Al, Al/Zn) and improves building properties. An important parameter for this pre-treatment is the concentration of NaOH. The use of a solution below 0.1 mol/L NaOH may not be effective. Due to the seasonality and heterogeneity of MSWIBA, to select the optimal concentration of the NaOH solution, a specific batch of material should be previously tested, mainly for its content of Al and Zn, to provide for the proper amount of NaOH as indicated by the stoichiometry in the given reactions (1) and (2) [18].

The course and effectiveness of the NaOH pre-treatment process depends not only on the concentration of NaOH, but also on the temperature and duration of the process [18,19].

To improve the pre-treatment, the stabilization time can be extended. However, some researchers have shown that the compressive strength can be increased with the appropriate ratio of aggregate to MSWIBA and if water pre-treatment is used [18].

In previous work, researchers investigated how different mass ratios of MSWIBA to aggregate values influence the compressive strength of cement [10]. The best compressive strength value was obtained for a MSWIBA/aggregate equal to 10%; however, any mixture of MSWIBA and aggregate showed better values than without MSWIBA. In normal circumstances, cement setting comes to strength standard in 28 days. It is estimated that 75% of the cement reacts during the maturation period. In the MSWIBA case, the setting takes longer, wherein after 90 days or more, compressive strength will probably be increase [20,21].

In the present work, the aim is to check how leachability in different environments (acid, alkali, aggressive, and normal) changes for mortars prepared with different types of MSWIBA. In particular, the mortars were prepared with addition of MSWIBA before valorization, after valorization, and after NaOH pre-treatment.

### 1.2. Life Cycle Assessment (LCA)

Every time a new process is proposed for recycling or recovery a waste stream, it is almost compulsory to check whether the new process effectively offers environmental improvements compared to conventional processes by performing a Life Cycle Assessment (LCA) [22].

Several authors have attempted to carry out an LCA of various MSWIBA management methods and the results indicate that the use of slag after waste incineration is associated with a positive environmental impact [23,24,25].

Results from one author noted up to 19% cost savings from waste-derived alkali-activated mortar in comparison to conventional alkali-activated mortar. Authors predict that chemically modified waste-derived activators are a promising alternative for improving the environmental performance of alkali-activated materials if their usage also reduces or replaces the need for conventional alkali-activators [25].

This study aims to provide a preliminary evaluation of the environmental impacts or benefits of using NaOH treatment for the alkali activation of MSWIBA by LCA.

## 2. Materials and Methods

### 2.1. Materials

**Figure 2:**
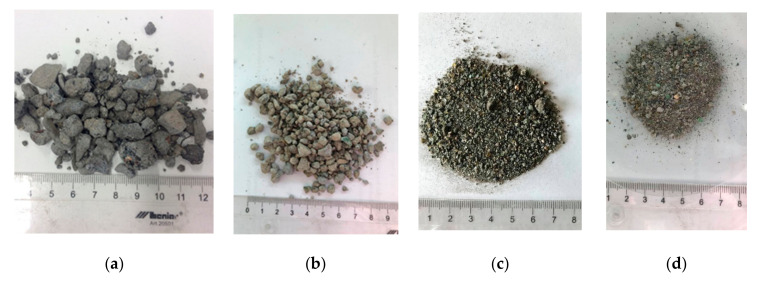
(**a**) Crude MSWIBA–MSWIBA before valorization; (**b**) MSWIBA after valorization; (**c**) 0–2 mm fraction; (**d**) MSWIBA after alkali activation (NaOH pretreatment).

MSWIBA were sampled from a MSW incineration plant before (Figure 2a) and after valorization (Figure 2b). The MSWIBA after valorization was ground to 0–2 mm in a hammer mill (Figure 2c). Then, the MSWIBA was flooded with NaOH and kept at a constant temperature of 55 °C for 3 h. The proportion was five to one by weight (five parts NaOH and one part MSWIBA). To completely remove the NaOH, the MSWIBA was washed several times with water until the pH was close to neutral (Figure 2d).

### 2.2. Methods

The samples of MSWIBA before and after the valorization and after the alkali activation were tested to check their construction properties and environmental behavior, and was leaching compared to environmental standards. In addition, phytotoxicity tests were carried out.

#### 2.2.1. Bottom Ash Characterization

For each sample, the following characterizations were performed. Moisture was determined in accordance with PN-Z-15008-02: 1993; total carbon (TC) in accordance with PN-EN 15407: 2011; total sulfur (S) in accordance with PN-ISO 351: 1999; chlorine (Cl) in accordance with PN-ISO 587: 2000; heavy metals in accordance with PN-EN 16174: 2012, PN-EN ISO 11885: 2009. The water extract was prepared in accordance with the PN-EN 12457-2: 2006 standard, and selected components were measured in accordance with the PN-ISO 9964-2/Ak: 1997 standard. Table 1 shows standards and methods of researches.

#### 2.2.2. Construction Properties 

Building mortars were produced. Thirty percent of the MSWIBA was used for each of the mortars as a substitute for CEM I 42.5R cement. Mortar composition was: 135 g of MSWIBA, 315 g CEM I 42.5R, 225 g of water, and 1350 g of sand, according to the PN-196 standard. The mortars were made in accordance with the PN-EN 480-1 standard [26,27]. Mortars, in the form of bars with dimensions of 4 × 4 × 16 cm were put into water for 28 days. The measurement was performed on an automatic press on which bending and compressive strength were measured.

#### 2.2.3. Leachability

To test the leachability, an extract from the blocks was prepared in accordance with the PN-EN 12457-4: 2006 standard [28]. Mortar samples were shaken for 24 h in an aqueous solution with the proportion 1:10. Acid, alkaline, and neutral solutions as well as an aggressive environment were prepared. The acidic reaction was made with the addition of HCl (acidic pH), the alkaline reaction with KCl (alkaline pH), the aggressive environment was prepared with H_2_CO_3_ (slightly acidic pH), and the neutral environment was prepared with distilled water (neutral pH). For comparison purposes, the samples were shaken in the same condition, at room temperature, and for the same time (24 h).

#### 2.2.4. Life Cycle Assessment

For the environmental comparison of the different valorization possibilities, a Life Cycle Assessment (LCA) was applied. The impact assessment method used was ReCiPe 2008, calculated with the aid of SimaPro 8.0.

There are many methodologies for LCA. One of them is ReCiPe, which is a methodology to bring together the endpoint and midpoint of environmental analysis (using multiple impact categories, discussed later). The most important goal of ReCiPe is to transform a long list of LCI (life cycle inventory) results into a limited number of indicators. These indicators express the relative environmental impact. The ReCiPe methodology defines two levels of indicators and each user can choose on which level they want the score:

○ Eighteen detailed waypoints that are relatively accurate but difficult to interpret;
○ Three simple to understand, but more imprecise endpoints:
  - for human health;
  - about the ecosystem;
  - for natural resources.

The user can choose between indicators and correctly interpret the indicators as part of a specific analysis. Another advantage of the methodology is the possibility of weighing the final results and presenting simplified values for the entire analysis (e.g., for three categories of damage). We express the values in DALY units for human health, species.yr for ecosystems, $ for resources.

The endpoint characterization factors used in ReCiPe can be described as follows:

1. Human health, expressed as the number of years life lost and the number of years lived disabled. These are combined as Disability Adjusted Life Years (DALYs), an index that is also used by the World Bank and WHO. The unit is years;
2. Ecosystems, expressed as the loss of species over a certain area, during a certain time. The unit is years;
3. Resources surplus costs, expressed as the surplus costs of future resource production over an infinitive timeframe (assuming constant annual production), considering a 3% discount rate. The unit is 2000US $.

Individual weights for given categories are implemented in the software, which is a tool for analysis, e.g., SimaPro, on which this study is based. They can also be changed if there is a need to modify the significance of individual components of the environmental impact categories. Program indicators were used in the presented analysis.

**Figure 3:**
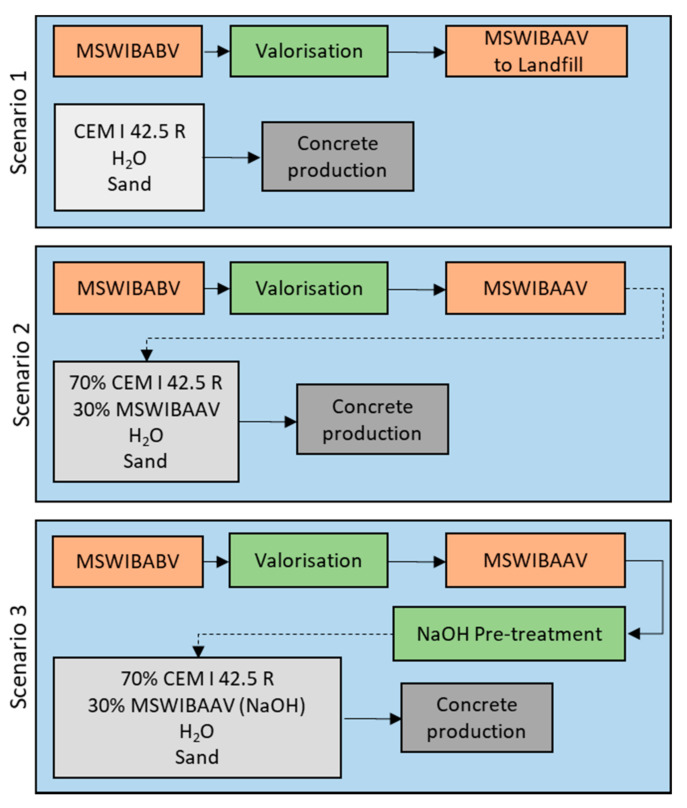
LCA assumptions of three different methods for MSWIBA treatment (MSWIBA**BV**–MSWIBA before valorization; MSWIBA**AV**–MSWIBA after valorization).

The goal was to analyze the environmental impact of three similar concrete blocks produced using three different scenarios of MSWIBA treatment. Scenario 1 includes the analysis of a standard concrete mix block, Scenario 2 includes the analysis of a concrete mix block with MSWIBA without NaOH treatment, and Scenario 3 includes the analysis of a concrete mix block with slag and NaOH treatment. The assumptions of the three scenarios are presented in Figure 3.

The study aim was to compare the environmental performances of the following production processes of one concrete block weighing 2.025 kg (i.e., the functional unit): (i) standard concrete mix, without the use of slag, with CEM I 42.5R cement; (ii) production of a block using MSWIBA slag after valorization as a replacement for 30% CEM I 42.5R; (iii) production of concrete mix by the assumptions made for one concrete block, which is the basis of the article. For production, we used 135 g MSWIBAAV, 315 g CEM I 42.5R, 225 g H_2_O, 1350 g sand, and 157.5 g NaOH (50%) for the MSWIBAAV pre-treatment process before making the mixture, after valorization.

The system boundaries include the process using basic materials in concrete production and using the BAAV as a part of raw material in scenarios 2 and 3. While they do not include the processes related to the slag valorization, they are the same in the three cases. The analysis does not include the energy consumption and consumption of other substances, or the waste flows or transportation during the production, because these are the same in the 3 scenarios. The analysis of Scenario 3 includes additional NaOH consumption, which is needed for the pre-treatment process. The analysis does not include the heat needed for the pre-treatment process because the assumption is that waste heat can be used from the factory where MSWIBA is produced, valorized, and pretreated. Primary data were used for the mixture composition. Secondary data were provided using the appropriate records of the Ecoinvent database.

## 3. Results

MSWIBA before valorization contains pollutants such as glass (18%), porcelain (0.5%), and metals (0.1%). MSWIBAAV is metal-free because of the valorization process, in which ferrous and non-ferrous metals are separated.

NaOH pretreatment was performed to etch the contact surface of MSWIBA. Alkali soaking increases the contact surface with the binder.

All three bottom ash samples were light grey and non-dusting, without a characteristic smell.

### 3.1. Botton Ash Characterization

Table 2 shows selected parameters for the three types of MSWIBA. Moisture content has an effect on concrete hydration, while carbon has an effect on surface aesthetics. Sulfur causes sulfate corrosion, while chloride causes reinforcement corrosion. A high content of heavy metals has negative effects on environment [12].

MSWIBA before valorization, MSWIBA after valorization, and after NaOH research present that:

- Carbon does not change in all samples;
- Sulphur decreases from 0.48% in MSWIBA before valorization to 0.20% in MSWIBA after valorization and 0.07% after NaOH pre-treatment;
- Chloride decreases from 0.41% in MSWIBA before valorization to 0.12% in MSWIBA after valorization and <0.01% after NaOH pre-treatment;
- Manganese decreases strongly after valorization (from 1178.35 ppm to 463.46 ppm) and less strongly after NaOH treatment (403.04 ppm);
- Nickel decreases after valorization, but does not decrease after NaOH treatment;
- Lead decreases after valorization, but less strongly after NaOH treatment;
- Cobalt decreases after valorization and after NaOH treatment;
- Chrome decreases after valorization and after NaOH treatment;
- Copper decreases after valorization and decreases more after NaOH treatment.

The contents of the undesired compounds such as sulfur, chloride, and most heavy metals strongly decrease from samples before valorization to samples after NaOH pre-treatment.

In the case of samples after the valorization process, the removed content of the different substances is expected to be found in the other solid streams mechanically separated from the main one (the only one analyzed here), and likely in the recovered metal streams.

The further decrease observed for samples after the NaOH pre-treatment needs a more accurate study. The amount of each compound removed will be found in the exiting NaOH-rich liquid stream. Thus, a full characterization of this stream is needed in future experiments to perform a complete mass balance for each substance and to understand which kind of treatment can be applied at an industrial scale to comply with environmental regulations.

### 3.2. Building Research

Table 3 shows the results of strength tests on concrete bars prepared with 30% MSWIBA after valorization or 30% MSWIBA after NaOH pretreatment. The use of MSWIBA after valorization caused the material to swell. 

The mortars with MSWIBA after NaOH pre-treatment present slightly higher values of bending (+15%) and compressive (+1%) strength than mortars with MSWIBA after valorization.

The research indicates the necessity to carry out the slag valorization process. The lack of the valorization process causes the mixture to swell. The mixture swelling excludes MSWIBA from being implemented in the industry. Research shows that NaOH pre-treatment has a positive effect on the strength of the mixture. The NaOH treatment process may vary. The final effect of MSWIBA on the physicochemical properties is influenced by the concentration of NaOH, the NaOH ratio, the processing time, and the temperature of the process.

### 3.3. Polution Leachability

Table 4 shows the results of the leachability of Na, K, Li, Ca, and Ba from the tested mortars in various environments.

The results show that the leaching of pollutants depends mainly on the water environment’s pH. The lowest leachability was noticed in the case of a neutral environment in which distilled water was used. The greatest leachability was in aggressive and alkaline water environments. The leachability is also influenced by the alkali pre-treatment, where in the case of Ba, the leachability is lower for blocks with MSWIBA after NaOH pre-treatment, while it is rather constant for Li. Na leachability increase was noted for mortars with MSWIBA after NaOH pre-treatment, as is evident because the concentrated NaOH solution was used.

Unfortunately, in the environment, ideal laboratory conditions do not exist, but in Europe, the majority of soil is acidic. This translates into less leaching of contaminants when using bottom ash as a building material in comparison with an alkaline environment [28].

The results of the research indicate the need to study the leachability of pollutants through sequential extraction due to different bioavailability. Sequential extraction distinguishes between water-soluble, ion-exchange, hydroxide, organic-related, and residual fractions. In the case of the techniques usually used in research, i.e., with distilled water, the leachability is the lowest. The low extraction effect is due to the lack of buffering capacity and high solubility of organic compounds. The ion exchange fraction uses the adsorption and desorption process. The hydroxide fraction takes advantage of the reducing properties of the compounds. The carbonate fraction leaches under conditions of lowered pH, e.g., acid rain and oxygen deficiency [29].

### 3.4. LCA

The analysis of the three scenarios was made based on assumptions using SimaPro software ReCiPe EndPoint V1.12/Europe ReCiPe H/A method in three general impact categories: human health, ecosystems, and resources.

The benefits associated with the use of MSWIBA are included in the data used from the Ecoinvent database. The results of the LCA analysis were compared in Table 5 and should be used as a rough estimation of the environmental impact of ash in concrete production. It shows that the use of slag from municipal waste incineration processes has a positive effect on the environment, despite the low environmental cost of using energy and substances for valorization.

In all impact categories, the results for the concrete block without the use of slag are the highest. The results showed that the environmental burden of using NaOH is higher compared to the slag-based block without NaOH pre-treatment, because the addition of NaOH is harmful to the environment compared to the valorization process itself. It is obvious, but the load values for most of the impact categories for scenarios 2 (s2) and 3 (s3) are the maximum difference in the human health category: s2 ~50% less DALY and s3 ~30% less DALY. s2 around is 45% less $ in the resources category, while the impacts of s1 and s3 are similar (around 1.5% lower impact for NaOH treatment). In the ecosystem category, the impact is 35% for s3 in comparison to s1, and is more than 50% lower for s2.

The LCA in this case is only an estimation to provide first view of the environmental aspects. In comparison to different LCA for using slag or fly ash in concrete production, we can observe that the environmental impact in most of the categories is more environmentally friendly than conventional production [30].

Future study including more specific data is required to check all parameters and variables. However, the first positive results come from this study. In the sensitivity analysis, the main contributor in the presented case with NaOH treatment is NaOH. If we change the production process of NaOH, the results are similar. Table 6 presents the sensitivity analysis for three different NaOH production processes used in Scenario 3 for NaOH pre-treatment (the database for the rest of inputs is the same).

In the LCA, the average production process of NaOH was used, but between NaOH production process, the difference is the highest for the human health category, around 7% in Process 2 compared to the other processes (1 and 3).

## 4. Discussion

Samples of MSWIBA before and after valorization and after NaOH pre-treatment were characterized. It a decrease in heavy metal contents after valorization and a further decrease after NaOH pre-treatment were noted.

The addition of 30% MSWIBA after NaOH pre-treatment to concrete mix slightly improves the bending and compressive strength of the concrete obtained.

Leaching test results of concrete mixes obtained by adding 30% of MSWIBA before or after valorization or after NaOH pre-treatment show that, in general, the NaOH pre-treatment allows for pollutant reduction.

Therefore, this article proposes an initial LCA analysis for the analyzed scenarios with or without the use of NaOH for pre-treatment in order to examine the environmental impact of the proposed slag valorization method in comparison to standard concrete block production. The benefits of the slag process with NaOH pre-treatment are not only linked with production, but are also relevant later during the use phase, because the leachability of the heavy metals can be reduced.

Despite the use of NaOH, concrete mix created without slag has an almost twice as great environmental burden as the other two methods: with the use of slag with and without NaOH treatment. It should also be emphasized that this preliminary analysis does not take into account the positive benefits related to the lack of leachability of heavy metals into the environment and the increased strength of the concrete blocks, which far outweigh the negative effects of using NaOH in the pre-treatment process [30,31].

The slight negative environmental impact of using NaOH can be reduced by reusing it after the process and/or reusing it in another technological process, or by reducing the concentration from the 50% concentration used in this analysis. It seems obvious that post-process NaOH is a product that can be reused; hence, its environmental impact on the process itself after pre-treatment will be beneficial. According to the environmental analysis, the use of slag in the production of concrete mix gives a positive result and additionally, thanks to NaOH, we can reduce the leaching of heavy metals during the use of the material in construction and increase the strength and durability of the concrete, which presents another positive impact on the environment.

## 5. Conclusions

The conducted research has an impact on increasing social awareness in the field of waste management and climate change. Secondary waste recycling reduces negative anthropogenic impacts on the environment. The basic research conducted has implementation potential. Implementation can take place in an incineration plant or a separate installation, e.g., an installation for the production of concrete. MSWIBA can be used for the production of paving stones or industry halls.

The NaOH pre-treatment research is relatively new in the market. Examples and detailed NaOH pre-treatment are described in reference [18]. Alkaline processing affects the immobilization of heavy metals and improves the quality of MSWIBA, which translates into the final strength of the mixture.

The different presented natures of the water environment were intended to imitate real conditions. The test with distilled water is the reference test. There are different types of soil in the world. Soils are distinguished on the basis of pH: strongly acidic (pH < 4.5), acidic (pH 4.5 > 5.5), slightly acidic (pH 5.6–6.5), neutral (pH 6.6–7.2), and alkaline (pH > 7.2). If concrete is placed in soil, the soil’s pH will have an effect on the leachability of contaminants. The research shows the most favorable pH for the leaching of pollutants from the product.

Despite the fact that NaOH in high density seems to have high impact on the environment, the benefit of using it is less leaching from the concrete block. Additionally, the NaOH can be reused in different processes of waste management, for example, to increase biogas production [32], to eliminate heavy metals in biochar derived from swine manure or hydrogen production [33], or even for anaerobic digestion, where amounts of H_2_O_2_ and moderate NaOH could improve the processes [34]. The NaOH could be also used in sewage disinfection to shorten the lifetime of *E. coli* bacteria [35]. This reuse of NaOH can decrease the environmental impact of using this substance for pre-treatment of slag before its use in concrete production.

## Figures and Tables

**Table 1:** Standards and methods of researches used in study.

Parameter	Standard
Moisture (M)	PN-Z-15008-02: 1993
Total Carbon (TC)	PN-EN 15407: 2011
Total Sulfur (S)	PN-ISO 351: 1999
Chlorine (Cl)	PN-ISO 587: 2000
Heavy Metals	PN-EN 16174: 2012, PN-EN ISO 11885: 2009
Water Extract (Leeachability)	PN-EN 12457-2: 2006
Sodium, Potassium, Lithium, Calcium, Bar (Na, K, Li, Ca, Ba)	PN-ISO 9964-2/Ak: 1997

**Table 2:** Selected pollution in MSWIBA before and after valorization, and after the NaOH pre-treatment.

Parameter	Symbol	Unit	MSWIBA before Valorization		MSWIBA after Valorization		After NaOH Pre-Treatment	
			Average Result	Result Standard Deviation	Average Result	Result Standard Deviation	Average Result	Result Standard Deviation
Moisture	M	%	4.18	0.14	8.65	0.15	n.d. **	n.d. **
Total Carbon	TC	%	0.85	0.46	0.85	0.43	0.84	0.38
Sulphur	S	%	0.48	0.16	0.20	0.09	0.07	0.01
Chloride	Cl^−^	%	0.41	0.12	0.12	0.04	<0.01	n.d. **
Manganese	Mn	ppm	1178.35	43.83	463.46	21.64	403.04	17.36
Cadmium	Cd	ppm	LOQ *	n.d. **	LOQ *	n.d. **	LOQ *	n.d. **
Nickel	Ni	ppm	45.99	5.78	10.41	2.96	11.68	2.85
Lead	Pb	ppm	379.50	12.69	176.47	10.36	154.61	10.97
Cobalt	Co	ppm	13.58	2.65	6.18	1.85	2.92	0.34
Chrome	Cr	ppm	1618.31	39.26	49.32	4.36	20.72	2.39
Copper	Cu	ppm	2954.00	50.36	2484.10	42.36	1192.10	42.44

* The limit of quantification; ** no data.

**Table 3:** Strength test results. MSWIBA accounts for 30% of cement by weight.

Type of Cement in Mortar	28-Day Bending Strength of Mortar (MPa)	*28-Day Bending Strength Standard Deviation*	28-Day Compressive Strength of Mortar (MPa)	*28-Day Compressive Strength Standard Deviation*
30% MSWIBA after valorization and CEM I 42.5R	4.60	*0.23*	25.90	*1.90*
30% MSWIBA NaOH pretreatment and CEM I 42.5R	4.65	*0.70*	29.92	*2.90*

**Table 4:** Pollution leachability of Na, K, Li, Ca, and Ba from mortars prepared with 30% MSWIBA before or after valorization or after NaOH pretreatment in acidic, neutral, alkaline, and aggressive environments.

		Na	K	Li	Ca	Ba
Mortar with MSWIBA before valorization/acid environment	**Result**	**21.97**	**24.67**	**0.24**	**41.45**	**10.34**
	*Standard Deviation*	*1.44*	*1.52*	*0.02*	*0.58*	*0.98*
Mortar with MSWIBA after valorization/acid environment	**Result**	**23.45**	**24.39**	**0.22**	**36.76**	**10.53**
	*Standard Deviation*	*0.74*	*0.60*	*0.01*	*0.97*	*0.79*
Mortar with NaOH pre-treatment MSWIBA/acid environment	**Result**	**32.63**	**18.74**	**0.22**	**39.45**	**9.71**
	*Standard Deviation*	*1.02*	*0.63*	*0.01*	*0.55*	*0.34*
Mortar with MSWIBA before valorization/aggressive environment	**Result**	**15.52**	**29.56**	**0.33**	**74.19**	**13.66**
	*Standard Deviation*	*0.39*	*0.55*	*0.01*	*0.82*	*0.68*
Mortar with MSWIBA after valorization/aggressive environment	**Result**	**15.53**	**28.47**	**0.31**	**65.51**	**13.01**
	*Standard Deviation*	*0.22*	*0.41*	*0.00*	*0.40*	*0.60*
Mortar with NaOH pre-treatment MSWIBA/aggressive environment	**Result**	**31.51**	**21.82**	**0.31**	**73.45**	**10.68**
	*Standard Deviation*	*1.30*	*0.94*	*0.01*	*0.86*	*0.56*
Mortar with MSWIBA before valorization/neutral environment	**Result**	**11.83**	**13.67**	**0.15**	**16.57**	**8.62**
	*Standard Deviation*	*0.13*	*0.71*	*0.00*	*0.31*	*0.73*
Mortar with MSWIBA after valorization/neutral environment	**Result**	**12.60**	**15.08**	**0.15**	**16.30**	**8.59**
	*Standard Deviation*	*0.75*	*0.62*	*0.00*	*0.33*	*0.46*
Mortar with NaOH pre-treatment MSWIBA/neutral environment	**Result**	**17.80**	**14.94**	**0.16**	**17.90**	**8.66**
	*Standard Deviation*	*0.38*	*0.30*	*0.00*	*0.41*	*0.26*
Mortar with MSWIBA before valorization/alkaline environment	**Result**	**30.50**	n.d. *	**2.36**	**49.97**	n.d. *
	*Standard Deviation*	*1.33*	n.d. *	*0.02*	*0.35*	n.d. *
Mortar with MSWIBA after valorization/alkaline environment	**Result**	**34.07**	n.d. *	**2.37**	**50.10**	n.d. *
	*Standard Deviation*	*0.40*	n.d. *	*0.22*	*0.73*	n.d. *
Mortar with NaOH pre-treatment MSWIBA/alkaline environment	**Result**	**44.41**	n.d. *	**2.32**	**54.54**	n.d. *
	*Standard Deviation*	*0.56*	n.d. *	*0.30*	*0.58*	n.d. *

n.d. * no data.

**Table 5:** Results of LCA for different scenarios of concrete mix production.

Impact Category	Unit	Scenario 1	Scenario 2	Scenario 3
Human health	DALY	6.99 × 10^−07^	3.41 × 10^−07^	4.99 × 10^−07^
Ecosystems	species.yr	3.40 × 10^−09^	1.61 × 10^−09^	2.24 × 10^−09^
Resources	$	7.02 × 10^−03^	3.89 × 10^−03^	6.92 × 10^−03^

**Table 6:** The sensitivity analysis of NaOH production processes in Scenario 3.

Impact Category	Unit	Process 1 Sodium Hydroxide (50% NaOH)	Process 2 Sodium Hydroxide, from Amalgam Technology (50% NaOH)	Process 3 Sodium Hydroxide, from Concentrating Membrane (50% NaOH)
Human health	DALY	4.99 × 10^−07^	5.20 × 10^−07^	4.83 × 10^−07^
Ecosystems	species.yr	2.24 × 10^−09^	2.29 × 10^−09^	2.19 × 10^−09^
Resources	$	6.92 × 10^−03^	7.12 × 10^−03^	6.70 × 10^−03^

## Data Availability

Not applicable.
